# Seronegative Ocular Myasthenia Gravis in an Older Woman With Transient Dizziness and Diplopia

**DOI:** 10.7759/cureus.27826

**Published:** 2022-08-09

**Authors:** Naho Yoshioka, Yumi Naito, Keisuke Sano, Chiaki Sano, Ryuichi Ohta

**Affiliations:** 1 Family Medicine, Shimane University, Izumo, JPN; 2 Community Care, Unnan City Hospital, Unnan, JPN; 3 Otolaryngology, Unnan City Hospital, Unnan, JPN; 4 Community Medicine Management, Shimane University Faculty of Medicine, Izumo, JPN

**Keywords:** general medicine, dizziness, nystagmus, diplopia, hospital, community, rural, older, seronegative, myasthenia gravis

## Abstract

Myasthenia gravis (MG) is a neuromuscular junction disease caused by an autoimmune response against cholinergic receptors. The challenge in diagnosing MG in older patients is the variety of symptoms and clinical manifestations. Clinical reasoning, precise history, and physical examination leading to a logical diagnosis should be performed to diagnose seronegative MG. We report a case of seronegative MG with the chief complaint of dizziness in a 91-year-old female. Despite the complicated clinical course, continuous clinical reasoning and testing can lead to appropriate diagnosis and treatment. As the dizziness symptoms in this older patient could not be explained by chronic or peripheral symptoms alone, ocular MG was considered as a possible diagnosis based on her history and physical examination findings. Appropriate diagnosis of seronegative ocular MG reactivated older patients with a good quality of life. In community medicine, where the behavior of elderly patients varies, it is important to improve the accuracy of diagnosis and treatment through appropriate history and physical examination, which will lead to longer home life in older patients.

## Introduction

Myasthenia gravis (MG) is a neuromuscular junction disease caused by autoimmunity to anticholinergic receptors [1]. This disease affects all neuromuscular parts of the human body [1]. Typical symptoms include eyelid drooping, arm and leg weakness, and difficulty in standing. Eyelid drooping is a common symptom with a prevalence of 50% [2]. MG symptoms fluctuate within a day and gradually exacerbate from morning to evening [1,2]. Disease progression can be fatal when symptoms such as dysphagia and respiratory failure occur [3]. Therefore, appropriate diagnosis and treatment are required. The main treatment options include acetylcholine esterase inhibitors, steroids, and immunosuppressants. Thymomas co-occur in 15% of patients with MG, and tumor resection may alleviate the symptoms of MG [4]. For an effective diagnosis of MG, clinicians should be alert to various symptom changes during the clinical course and perform prompt clinical tests to clarify the diagnosis.

The challenge in diagnosing MG in older patients is the variety of symptoms and clinical manifestations. The diagnosis of MG should be based on clinical symptoms and tests specific to the stimulation of neuromuscular junctions [5]. In addition to clinical symptoms, edrophonium infusion and ice pack tests are useful for diagnosing MG [1]. While repetitive stimulating neuromuscular tests can be performed for diagnosis, their performance can depend on the medical facilities [1]. Moreover, MG among older patients can show various symptoms in addition to typical symptoms, such as dizziness, gloss fatigue, appetite loss, and headaches [5,6]. However, laboratory tests for the detection of autoantibodies have limitations [7]. While detecting anti-acetylcholine receptors and anti-muscarinic receptor antibodies is important for diagnosis, seronegative MG has recently become prevalent, especially among older patients [7].

Thus, the diagnosis of seronegative MG requires clinical reasoning, precise medical history, and physical examination, leading to a logical diagnosis. We report a case of seronegative MG with the chief complaint of dizziness in a 91-year-old woman. The clinical course is complicated; however, continuous clinical reasoning and testing can lead to an appropriate diagnosis and treatment. This case report discusses the difficulty in diagnosing MG and practical ways to diagnose seronegative MG in rural hospitals.

## Case presentation

A 91-year-old woman was admitted to a rural community hospital with the chief complaint of dizziness. One month before admission, she experienced abnormalities in her right eye, including swelling around the eye while in a sitting position that improved in the supine position and wobbling to the right. She was only able to walk with the aid of a walker. Three days before admission, the rotatory dizziness resolved within one minute. Two days before admission, she visited an otolaryngologist and was diagnosed with peripheral vertigo. On the day of admission, she was transferred to the emergency room because she became dizzy and could not stand up from a lying position. The patient had a medical history of severe aortic stenosis, sigmoid colon cancer with endoscopic resection, chronic cardiac failure, type 2 diabetes mellitus, deep vein thrombosis, and anxiety disorder. Her medications included rosuvastatin (2.5 mg), rabeprazole (10 mg), telmisartan (40 mg), isosorbide (20 mg), apixaban (2.5 mg), vildagliptin (50 mg), arotinolol (5 mg), alprazolam (0.4 mg), and betahistine (6 mg).

On arrival, the patient was alert with a blood pressure of 162/97 mmHg, a pulse of 82 beats per minute, a respiratory rate of 16 breaths per minute, SpO_2_ of 95% (room air), and a temperature of 36.5°C. Physical examination revealed normal pupils with light reflexes, normal extraocular movements, and no abnormal neurological findings. Nystagmus was induced from the supine position to the left lateral position, which was in the right rotatory position. On admission, magnetic resonance imaging (MRI) was performed with no high signals observed in the brainstem areas or obvious occupying lesions compressing the intracranial oculomotor nerve. Laboratory tests performed on admission showed a vitamin B1 level of 22 ng/mL; thus, oral vitamin B1 supplementation was initiated (Table 1).

**Table 1:** Initial laboratory data of the patient. HBs: hepatitis B surface antigen; HBc: hepatitis B core antigen; HCV: hepatitis C virus; S/CO: sample-to-cut-off ratio; SARS-CoV-2: severe acute respiratory syndrome coronavirus 2

Marker	Level	Reference
White blood cells	8.30	3.5–9.1 × 10^3^/μL
Neutrophils	63.9	44.0–72.0%
Lymphocytes	27.7	18.0–59.0%
Monocytes	6.9	0.0–12.0%
Eosinophils	0.6	0.0–10.0%
Basophils	0.9	0.0–3.0%
Red blood cells	4.92	3.76–5.50 × 10^6^/μL
Hemoglobin	14.8	11.3–15.2 g/dL
Hematocrit	44.0	33.4–44.9%
Mean corpuscular volume	89.5	79.0–100.0 fL
Platelets	17.0	13.0–36.9 × 10^4^/μL
Erythrocyte sedimentation rate	10	2–10 mm/hour
Total protein	7.6	6.5–8.3 g/dL
Albumin	3.9	3.8–5.3 g/dL
Total bilirubin	0.9	0.2–1.2 mg/dL
Aspartate aminotransferase	21	8–38 IU/L
Alanine aminotransferase	11	4–43 IU/L
Alkaline phosphatase	79	106–322 U/L
γ-Glutamyl transpeptidase	19	<48 IU/L
Lactate dehydrogenase	195	121–245 U/L
Blood urea nitrogen	15.5	8–20 mg/dL
Creatinine	0.63	0.40–1.10 mg/dL
Estimate glomerular filtration rate	65.1	>60.0 mL/min/1.73m^2^
Serum Na	139	135–150 mEq/L
Serum K	3.2	3.5–5.3 mEq/L
Serum Cl	101	98–110 mEq/L
Serum Ca	9.3	3.5–10.3 mg/dL
Serum P	3.7	0.2–1.2 mg/dL
Serum Mg	1.7	1.8–2.3 mg/dL
Creatine kinase	13	56–244 U/L
C-reactive protein	0.25	<0.30 mg/dL
Thyroid-stimulating hormone	0.95	0.35–4.94 μIU/mL
Free T4	1.1	0.70–1.48 ng/dL
Vitamin B1	22	21.3–81.9 pg/mL
Folic acid	4.5	>4.0 ng/mL
Immunoglobulin G	1227	870–1,700 mg/dL
Immunoglobulin M	71	35–220 mg/dL
Immunoglobulin A	337	110–410 mg/dL
HBs antigen	0	IU/mL
HBs antibody	17.57	mIU/mL
HBc antibody	0.00	S/CO
HCV antibody	0.00	S/CO
Syphilis treponema antibody	0.00	S/CO
SARS-CoV-2 antigen	Negative	
Urine test		
Leukocyte	Negative	
Nitrite	Negative	
Protein	Negative	
Glucose	Negative	
Urobilinogen	Negative	
Bilirubin	Negative	
Ketone	Negative	
Blood	Negative	
pH	7.0	
Specific gravity	1.014	
Fecal occult blood	Negative	

The transient nystagmus persisted. Symptoms of diplopia appeared on day 15 of hospitalization. Physical examination revealed an adduction impairment in the left eye. The patient was able to converge on her eyes. Symptoms of diplopia with positive physical findings were reversible and exacerbated daily in the afternoon and evening. Reexamination of brain MRI did not show any central abnormalities. Her appetite loss gradually worsened, and we suspected MG; however, anti-acetylcholine receptor and anti-muscarinic receptor antibodies were negative. Chest computed tomography (CT) showed no thymoma. On day 27 of hospitalization, diplopia and bilateral rightward nystagmus were observed with increasing intensity and variable fatigue. In addition, as she developed ptosis, an ice pack test was administered to her eyes, improving the objective findings and subjective symptoms of ptosis.

**Figure 1:**
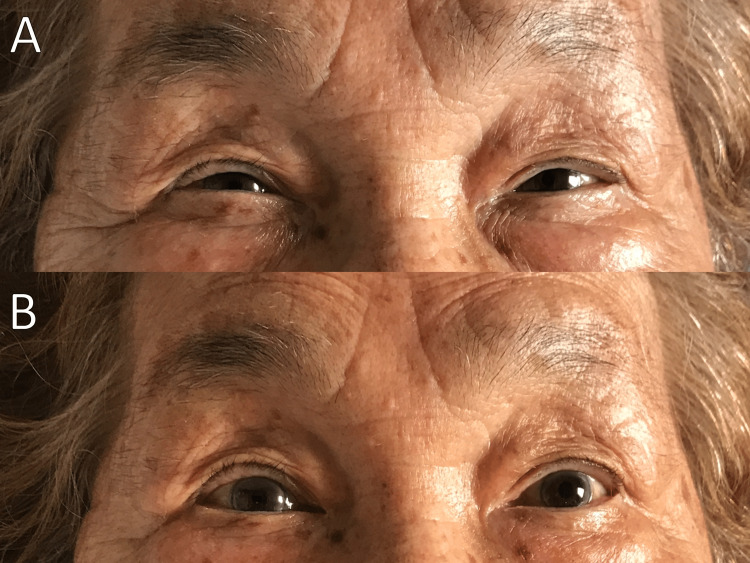
Change in the patient’s face during the edrophonium test. (A) Before edrophonium infusion. (B) Five minutes after edrophonium infusion.

Otolaryngology examinations, including the vestibular function test (VFT) and eye tracking test (ETT), performed on day 27 of hospitalization revealed nystagmus induction, negative for caloric and possible opsoclonus/ocular flutter, indicating both central and peripheral etiologies of nystagmus. Laboratory test results for antinuclear antibodies were negative on day 35 of hospitalization. An edrophonium test was performed on day 37 of hospitalization to diagnose seronegative MG. The edrophonium test transiently alleviated eyelid drooping and nystagmus (Figure 1).

Thus, the patient was diagnosed with seronegative MG. Prednisolone (PSL) (30 mg) was initiated on day 38 of hospitalization. On hospitalization day 39, pyridostigmine was started for symptom control, alleviating her decreased appetite, dizziness, nystagmus, and ptosis. On day 41 of hospitalization, we confirmed that she had experienced no side effects of pyridostigmine, and the dose was increased to three tablets per day. On day 43 of hospitalization, tacrolimus (1 mg) was added, and the PSL dose was reduced to 20 mg. Her symptoms completely improved, and she was discharged home on the 46th day of hospitalization with a prescription of PSL (20 mg), pyridostigmine (180 mg), and tacrolimus (1 mg).

## Discussion

This case report describes the case of an older woman who presented with vertigo symptoms and was finally diagnosed with seronegative MG based on the edrophonium test results. The patient tested negative for autoantibodies; however, her symptoms improved after treatment with PSL, pyridostigmine, and tacrolimus. This case presentation suggests that seronegative MG should be included in the differential diagnosis of patients with a chief complaint of long-lasting dizziness, even if the patient has various symptoms, such as medial longitudinal fasciculus (MLF) syndrome or peripheral vestibular neuropathy. Observing the daily changes in ptosis and other symptoms is also important.

Regarding dizziness, one of the common symptoms regarded as an indeterminate complaint among older patients, MG should be included in the differential diagnosis through appropriate history and physical examination [1]. In our case, bilateral nystagmus occurred at the time of presentation and progressed throughout the clinical course. Otolaryngological examination showed negative nystagmus and caloric results, suggesting the possibility of both central and peripheral vertigo, which could be caused by MLF syndrome and peripheral vestibular neuropathy [8]. However, MLF syndrome and peripheral vestibular neuropathy do not show daily changes in the clinical course; therefore, it is necessary to differentiate these diseases from MG.

MG is often associated mainly with myasthenic symptoms of the peripheral musculature but may also show central symptoms. Ocular MG in the elderly may manifest as ocular muscle abnormalities and peripheral neurological symptoms similar to central vertigo [8,9]. However, these symptoms caused by other diseases often show little diurnal variation and may be considered as a differentiating factor from MG [5]. In the present case, diurnal fluctuations were observed during the whole clinical course. In addition, fatigue of the bilateral external ocular muscles due to ocular MG was observed at different times, leading us to suspect a seronegative MG. Dizziness is a common symptom in a geriatric care setting; however, when it is chronic and cannot be explained by only peripheral nerve disorder, it is necessary to consider the possibility of neuromuscular disease, including MG, even though typical serological tests for MG are negative.

The diagnostic process cannot rely solely on antibody testing to diagnose ocular MG in elderly patients. Our patient was negative for anti-acetylcholine receptor and anti-MuSK antibodies, suggesting the possibility of seronegative MG. Because only ocular symptoms were initially observed in this patient, it was thought that only the ocular muscles were affected. However, the patient developed symptoms other than ocular muscle involvement, including anorexia and a loss of motivation for rehabilitation. A previous study showed that ocular MG tends to be negative for anti-acetylcholine receptors and anti-MuSK antibodies [10]. In our case, because of her advanced age, the patient did not wish to undergo invasive examinations; thus, we could not perform evoked electromyography. We diagnosed the patient with seronegative MG and proceeded with treatment based on the edrophonium stress test results. After the start of treatment, the patient’s appetite increased, and her motivation for rehabilitation returned, suggesting that her symptoms of swallowing dysfunction and weakness improved. In medical care for older patients with dizziness, a prolonged period of scrutiny and follow-up may worsen long-term prognosis and quality of life [11]. To diagnose ocular MG effectively, general physicians need to detect the atypical clinical course of dizziness among older patients [12].

Although the diagnosis and treatment for ocular MG in community hospitals are challenging, it is feasible by using a comprehensive approach. The patient in this case presented with a chief complaint of dizziness and symptoms, such as loss of appetite and decreased activity, which are common in the elderly. Considering her appearance and complaints of symptoms that were not typical among older patients, we could identify the characteristic symptoms of ocular MG with diurnal variations. Appropriate history taking and physical examination can allow ambiguous symptoms of the elderly to be categorized for diagnosis and treatment [13]. In the future, appropriate medical history and physical examinations will be even more important in community medicine, where the behaviors of elderly patients are becoming more diverse [14].

## Conclusions

In this case, as the dizziness symptoms in this older patient could not be explained by chronic or peripheral symptoms alone, ocular MG could be considered a possible diagnosis patient developed fatigue symptoms and ptosis. Without ptosis on the examination, it could be hard to objectify the results of the edrophonium test. The diagnosis of seronegative ocular MG reactivated older patients with a good quality of life. In community medicine, where the behavior of elderly patients varies, it is important to improve the accuracy of diagnosis and treatment by appropriate history taking and physical examination, leading to longer home life in older patients.
